# Development of Vaginal In Situ Gel Containing ISN/HP-β-CD Inclusion Complex for Enhanced Solubility and Antifungal Efficacy

**DOI:** 10.3390/polym17040514

**Published:** 2025-02-16

**Authors:** Tarek Alloush, Gülsel Yurtdaş Kırımlıoğlu

**Affiliations:** 1 Institute of Health Sciences, Istanbul University, 34216 Istanbul, Türkiye; 2 Department of Pharmaceutical Technology, Graduate School of Health Sciences, Anadolu University, 26470 Eskişehir, Türkiye; 3 Department of Pharmaceutical Technology, Faculty of Pharmacy, Anadolu University, 26470 Eskişehir, Türkiye; gyurtdas@anadolu.edu.tr

**Keywords:** isoconazole nitrate, solubility enhancement, hydroxypropyl-β-cyclodextrin, spray drying, inclusion complexes, in situ gel

## Abstract

Many antifungal agents, including isoconazole nitrate (ISN), suffer from low aqueous solubility and inconsistent dissolution kinetics, which limit their therapeutic potential. To address these challenges, this study aimed to enhance the solubility and stability of ISN through the development of inclusion complexes with hydroxypropyl-β-cyclodextrin (HP-β-CD). HP-β-CD inclusion complexes were prepared using a spray-drying technique and characterized through phase-solubility studies, scanning electron microscopy (SEM), Fourier transform infrared spectroscopy (FT-IR), proton nuclear magnetic resonance (^1^H-NMR), and differential scanning calorimetry (DSC). The inclusion complex significantly improved ISN solubility, increasing from 0.5088 mg/mL to 3.6550 mg/mL. These complexes were incorporated into a thermosensitive, mucoadhesive in situ gel system using Pluronic^®^ F127 and hydroxypropyl methylcellulose (HPMC) to optimize vaginal drug delivery. The formulations were evaluated for gelation temperature, viscosity, swelling behavior, and pH, confirming their suitability for vaginal application. Antimicrobial studies demonstrated that the ISN/HP-β-CD gels exhibited superior activity against *Candida albicans*, *C. glabrata*, and *C. krusei* compared to ISN alone. In vitro release studies further revealed sustained drug release following Peppas-Sahlin kinetics, supporting enhanced bioavailability and prolonged therapeutic action. This study demonstrates that the ISN/HP-β-CD-loaded in situ gel system offers a promising and effective approach for improving the solubility, stability, and antifungal efficacy of ISN for the treatment of vaginal infections.

## 1. Introduction

Vaginal candidiasis, sometimes called vulvovaginal candidiasis, is a common vaginal mucosal infection that is mostly caused by the Candida species [1,2]. It is the second most frequent mucosal infectious condition after bacterial vaginosis. This condition affects a significant proportion of women worldwide, with more than 80% of women expected to experience a vaginal infection at least once in their lifetime [1]. Symptoms such as itching, burning, irritation, and abnormal discharge can severely impact the quality of life [3]. Additionally, recurrent vaginal candidiasis, affecting up to five percent of women, incurs substantial healthcare costs and poses a challenge for effective treatment [4]. Traditional vaginal therapies, including creams, ointments, and semi-solid gels, are commonly used but are associated with significant limitations such as leakage, messiness, and insufficient retention in the vaginal cavity, leading to reduced therapeutic efficacy and patient compliance [5].

**Figure 1:**
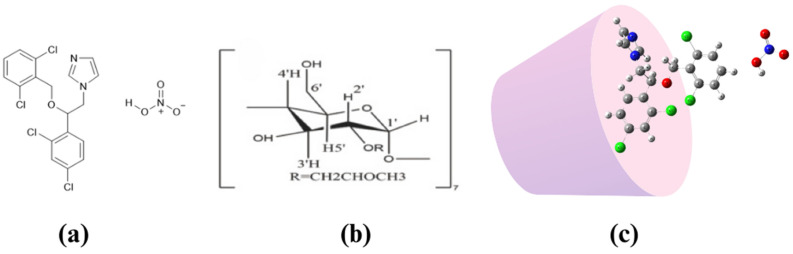
Chemical structures of (**a**) isoconazole nitrate (ISN), (**b**) hydroxypropyl-β-cyclodextrin (HP-β-CD), and (**c**) the proposed inclusion complex of ISN/HP-β-CD.

Isoconazole nitrate (ISN), a broad-spectrum antifungal agent from the azole class, is widely used for the treatment of vaginal infections [6,7]. Its strong activity against Candida species and Gram-positive bacteria makes it a preferred choice for managing vaginal candidiasis. However, ISN has low aqueous solubility and is highly sensitive to environmental factors such as light and temperature, which limits its bioavailability and overall therapeutic potential (Figure 1a). These formulation challenges necessitate innovative strategies to improve ISN’s solubility, stability, and retention in the vaginal cavity.

Recently, a lot of research has been conducted to increase the bioavailability of vaginal therapies. Cyclodextrins (CDs) are highly preferred because they efficiently increase solubility. CDs are cyclic oligosaccharides with a hydrophilic outer surface and a hydrophobic inner cavity, enabling them to form inclusion complexes with poorly water-soluble drugs (Figure 1b). These complexes encapsulate hydrophobic molecules, protecting them from environmental degradation while significantly enhancing their solubility and stability [8,9]. Among CD derivatives, hydroxypropyl-β-cyclodextrin (HP-β-CD) is particularly advantageous due to its superior water solubility, low toxicity, and high complexation efficiency compared to native β-CD [10,11]. HP-β-CD has demonstrated efficacy in improving the solubility and stability of a range of drugs, including antifungal agents like ISN. This makes it an ideal candidate for addressing ISN’s formulation challenges.

Vaginal in situ gelling systems present a promising platform for overcoming the limitations of traditional formulations [5]. These systems are liquid at room temperature, enabling easy administration, but undergo a sol-to-gel transition upon exposure to physiological conditions such as body temperature or vaginal pH [12,13]. This transformation enhances mucoadhesive properties [14], prolongs the residence time of the formulation in the vaginal cavity, and allows for sustained drug release. Such systems have been successfully utilized for a variety of therapeutic agents, including estriol and meloxicam, demonstrating improved efficacy and patient compliance [15,16].

Despite advancements, several gaps remain in the development of vaginal drug delivery systems. Many current formulations still suffer from rapid drug clearance, inadequate retention, and poor solubility of active agents. Furthermore, while cyclodextrins have been widely studied, their integration into thermosensitive vaginal in situ gels remains underexplored [17]. This study aimed to address these challenges by developing a thermosensitive, mucoadhesive in situ gel containing ISN/HP-β-CD inclusion complexes (Figure 1c). The objectives were to enhance ISN’s solubility and stability, improve its retention time in the vaginal cavity, and evaluate the formulation’s in vitro release, antimicrobial activity, and stability. By incorporating Pluronic^®^ F127 as a thermosensitive polymer and hydroxypropyl methylcellulose (HPMC) as a mucoadhesive agent, this study provides a novel therapeutic strategy for the effective treatment of vaginal infections [18].

## 2. Materials and Methods

### 2.1. Materials

Isoconazole nitrate (ISN; Deva Pharma, Istanbul, Turkey) and (2-Hydroxypropyl)-β-cyclodextrin (HP-β-CD; Sigma-Aldrich, Steinheim, Germany) were utilized in this study. Pluronic^®^ F127 (Poloxamer) and hydroxypropyl methylcellulose (HPMC) were also obtained from Sigma-Aldrich, Steinheim, Germany. Ethanol (99.5%) and methanol, both from Sigma-Aldrich, Germany, were used as solvents. Benzalkonium chloride was sourced from Fluka, Buchs, Denmark. The solution was prepared and diluted using ultrapure water (Merck, Darmstadt, Germany).

The Simulated Vaginal Fluid (SVF) was prepared using sodium chloride, potassium chloride, calcium hydroxide, bovine serum albumin, lactic acid, acetic acid, glucose, urea, glycerin, and distilled water, all sourced from Merck, Darmstadt, Germany, except glycerin, which was obtained from Doğa Pharma, Istanbul, Turkey.

*Candida albicans* (ATCC 90028), *Candida glabrata* (ATCC 90030), and *Candida krusei* (ATCC 6258) were grown in Sabouraud dextrose broth and cultured on Mueller-Hinton agar and Sabouraud dextrose agar, all supplied by Merck, Darmstadt, Germany. All chemicals and reagents used were of analytical grade and sourced from their respective suppliers.

### 2.2. HPLC Method Development and Validation

The HPLC system (Shimadzu 20-A, Shimadzu Corporation, Kyoto, Japan) consisted of a diode array detector, an autosampler, an interface, and a pump. A VDSpher 100 C18-E reversed-phase column (VDS optilab, Berlin, Germany) (250 × 4.6 mm, 5 μm) was used for analysis. The mobile phase was a 50:50 (*v*/*v*) mixture of methanol and potassium dihydrogen phosphate buffer. Samples were analyzed at a flow rate of 0.5 mL/min and monitored at a wavelength of 210 nm. The temperature of the column oven was maintained at 40 °C, and the injection volume was set at 20 μL. Each experiment was performed in triplicate to ensure accuracy and reliability.

### 2.3. Preparation of ISN/HP-β-CD Complex

A specified amount of ISN was dissolved in methanol, while HP-β-CD was dissolved in water, maintaining a 1:1 molar ratio. The two clear solutions were mixed using a magnetic stirrer to ensure homogeneity. The resulting mixture was dried using a spray dryer equipped with a 0.7 mm diameter spray head. The feed rate was set at 7 mL/min with an outlet temperature of 60 °C and a feed temperature of 100 °C. The drying process produced fine powder complexes, which were collected in the designated container for further analysis [19].

### 2.4. Structural and Physicochemical Characterization of ISN/HP-β-CD Complex

#### 2.4.1. Phase-Solubility Profile and Complexation Efficiency

An aqueous solution containing HP-β-CD in concentrations ranging from 2 × 10^−3^ to 20 × 10^−3^ M was prepared. Excessive ISN (20 mg) was added in a 3:1 ratio to a mixture of the prepared CD solution and ethanol. The mixture was shaken on a horizontal shaker at 25 °C to produce supersaturated solutions, which were allowed to reach equilibrium over 24 h. After equilibration, the solutions were filtered through a nylon filter (0.45 μm) and analyzed by HPLC. The resulting data, obtained from three independent experiments, were used to construct a phase solubility diagram. The type of diagram was classified according to the Higuchi phase solubility model [20].

#### 2.4.2. Physicochemical Characterization Tests of ISN/HP-Β-CD Inclusion Complex

ISN/HP-β-CD inclusion complexes were thoroughly characterized in vitro through investigations employing scanning electron microscopy (SEM), Fourier transform-infrared spectroscopy (FT-IR), proton nuclear magnetic resonance (^1^H-NMR), and differential scanning calorimetry (DSC). These measurements were performed for ISN, HP-β-CD, the physical mixture (ISN/HP-β-CD PM), and the ISN/HP-β-CD inclusion complex.

Differential scanning calorimetry (DSC; Shimadzu DSC-60, Shimadzu Corporation, Kyoto, Japan) was used to analyze the thermal behavior of the samples. Approximately 4 mg of each sample was sealed in aluminum pans, and the analysis was performed at a heating rate of 10 °C/min over a temperature range of 50–400 °C. Thermograms were recorded to evaluate the thermal properties.

Proton nuclear magnetic resonance (^1^H-NMR; Bruker, Bruker Corporation, Billerica, MA, USA) spectra were obtained for all samples using deuterated dimethyl sulfoxide (DMSO-d_6_) as a solvent to investigate the molecular interactions between the components.

Fourier transform-infrared spectroscopy (FT-IR; Shimadzu, Shimadzu Corporation, Kyoto, Japan) was employed to investigate the samples. Spectra were recorded in the range of 500–4000 cm^−1^ to identify functional group interactions and confirm complexation.

Scanning electron microscopy (SEM; Zeiss, Carl Zeiss AG, Oberkochen, Germany) was used to examine the surface morphology of the samples. The SEM images were analyzed to observe surface characteristics and structural differences.

### 2.5. Preparation of In Situ Gel Systems

In situ gel systems were prepared using a cold method to prevent gelling during the formulation process due to heat exposure. Pluronic^®^ F127 was the primary polymer used, and hydroxypropyl methylcellulose (HPMC) was incorporated into some formulations to evaluate its effect on the physicochemical properties of the gels.

The required amounts of Pluronic^®^ F127, HPMC, and benzalkonium chloride were accurately weighed and added to a beaker containing sufficient distilled water. The mixture was stirred continuously with a magnetic stirrer (2 mm diameter bar) at 500 rpm for 24 h at approximately 4 °C. To maintain a consistent low temperature, fresh ice molds were added to the setup every four hours.

The composition and codes of the prepared formulations are summarized in Table 1. This approach ensured uniform mixing and avoided premature gelation, resulting in stable formulations ready for further evaluation.

### 2.6. Physicochemical and Rheological Evaluation of In Situ Gel Formulations

The in situ gel formulations were evaluated for several parameters, including pH, gelation temperature, gelling capacity, swelling behavior, rheological properties, drug content (%), and antimicrobial efficacy. These characterizations were performed to ensure that the formulations met the desired performance criteria.

#### 2.6.1. pH Measurement

The pH of the prepared formulations was determined using a pH meter (Mettler Toledo, Mettler-Toledo International Inc., Columbus, OH, USA). Each measurement was performed in triplicate to ensure accuracy and reproducibility.

#### 2.6.2. Gelation Behavior and Stability

The gelling capacity of the formulations was evaluated by adding a drop of the formulation into a vial containing 2 mL of SVF at 37 °C. The gelation process was observed visually, considering both the gelation time and the duration of gel stability. The results were recorded and interpreted using the coding system outlined in Table 2, which categorizes the gelling behavior based on gelation and dissolution times [21].

#### 2.6.3. Swelling Behavior Assessment

To evaluate the swelling behavior of the in situ gel formulations, 1 mL of each formulation was placed in a dialysis membrane, sealed using a dialysis membrane sealer, and immersed in Simulated Vaginal Fluid (SVF) at 37 °C. After a predefined incubation period, the swollen gel was carefully removed, and excess surface fluid was gently blotted using filter paper. The mass of the swollen gel ($m_{s}$) was then recorded.

To determine the mass of the dried gel ($m_{d}$), the samples were left to dry completely under controlled conditions. The swelling percentage (SP) was calculated using the following equation [22]:

(1) $$SP=\left(\frac{\;m_{s}-m_{d}}{m_{d\;}}\right)\times 100,$$

where *m_s_* is the mass of swelled gel, *m_d_* is the mass of dried gel.

#### 2.6.4. Rheological and Viscosity Analysis

The rheological properties of the formulations were evaluated using a rheometer (Brookfield, AMETEK Brookfield, Middleboro, MA, USA) at two different temperatures: 25 °C and 37 °C. Changes in shear stress and viscosity versus shear rate were recorded to evaluate the flow behavior of the formulations at both temperatures.

#### 2.6.5. Drug Content Analysis

To determine the drug content (DC%) of ISN in the formulations, 100 mg of the prepared gel was dissolved in 1 mL of 50% methanol. The solution was then diluted to the required concentration with the same solvent and analyzed using a validated HPLC method.

#### 2.6.6. In Vitro Release Studies of In Situ Gel Formulations

A specific amount of the in situ gel formulation was carefully loaded into a dialysis bag, which was sealed and included a magnetic stirring bar to ensure consistent agitation during the in vitro release study, mimicking physiological conditions. The sealed dialysis bag was placed in a beaker containing 50 mL of SVF with a pH of 4.5 as the release medium. The experiment was performed at 37 ± 0.5 °C using a magnetic stirrer set to 100 rpm. At predetermined time intervals (0.25, 0.5, 1, 2, 3, 4, 6, 9, 12, 24, 48, 72, and 96 h), 1 mL samples were taken from the receptor medium and replaced with an equal volume of fresh SVF to maintain sink conditions. The collected samples were analyzed using a validated HPLC method. Drug release kinetics were evaluated using the DD Solver program to determine the release mechanism from the in situ gel systems.

#### 2.6.7. Antifungal Activity Against Candida Strains

Antimicrobial activity was assessed against *Candida albicans* (*C. albicans*, ATCC 90028), *Candida glabrata* (*C. glabrata*, ATCC 90030), and *Candida krusei* (*C. krusei*, ATCC 6258). The agar diffusion method, a widely used technique for evaluating microorganism sensitivity, was used. Petri dishes (90 mm diameter) were filled with 20–25 mL of Sabouraud dextrose agar under aseptic conditions and allowed to solidify. Then, 100 μL of yeast suspension, adjusted spectrophotometrically at 530 nm to match turbidity equivalent to 0.5 McFarland standard, was spread evenly over the agar surface.

Once the surface dried completely (approximately 10 min at 25 °C), 6 mm wells were created in the agar using a sterile cork borer. Each well was filled with 35 μL of the test formulation containing ISN at a concentration of 3 μg/mL. Plates were incubated at 37 °C for 24 h to allow for microbial growth and diffusion of the formulations. Zones of inhibition around the wells were measured to determine the antimicrobial efficacy of the in situ gel formulations.

## 3. Results

### 3.1. Validation of HPLC Method for ISN Quantification

**Figure 2:**
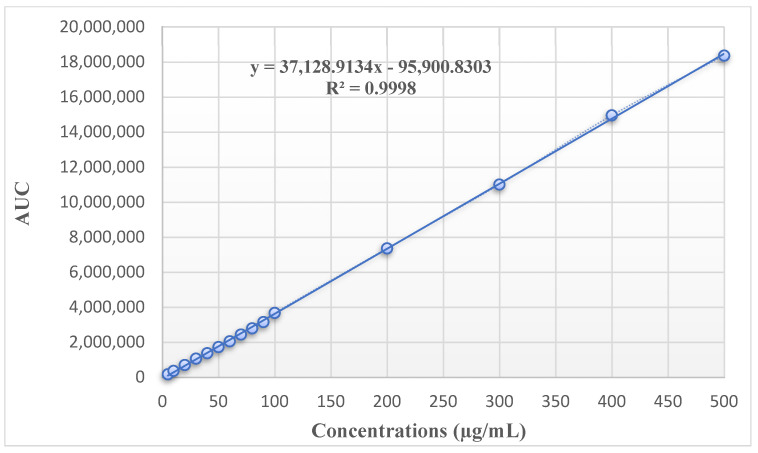
Calibration curve and linearity equation of ISN standard.

A calibration curve for ISN was prepared using the mobile phase as the diluent. The HPLC method, operating at a wavelength of 210 nm, exhibited excellent linearity across the concentration range of 20–500 μg/mL, with a correlation coefficient (r^2^) of 0.9998. The method was further validated, demonstrating that it meets all necessary criteria for alternative validation parameters. Figure 2 shows the calibration curve and the linearity equation for the ISN standard.

### 3.2. Structural and Physicochemical Characterization of ISN/HP-β-CD Complex

#### 3.2.1. Phase-Solubility Profile and Complexation Efficiency

Phase solubility studies provide essential information regarding the stability constant, complexation stoichiometry, and solubility enhancement of drug–CD inclusion complexes [11]. This method is widely used to evaluate how drug–CD complexation influences the solubility of poorly soluble drugs. Among the different inclusion types, the most common stoichiometry is a 1:1 drug–CD complex, where the stability constant (K1:1) quantifies the equilibrium between free and complexed species.

**Figure 3:**
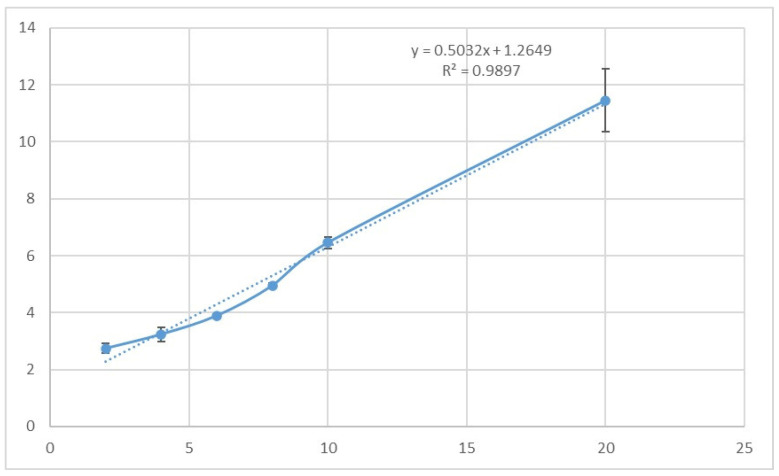
Phase solubility diagram of ISN/HP-β-CD.

The phase solubility plot (Figure 3) showed that the solubility of ISN increased proportionally with the concentration of HP-β-CD, confirming the formation of inclusion complexes. The plot was classified as “AL-type”, with a slope of 0.5032 and a strong correlation coefficient (R2 = 0.9897). The stability constant (Ks) for the ISN/HP-β-CD complex was calculated to be 803.52 M^−1^, which is within the acceptable range of 100–10,000 M^−1^, as reported in the literature, indicating stable complexation [23].

#### 3.2.2. Solubility of ISN/HP-β-CD Inclusion Complex

To evaluate the solubility enhancement achieved by complexation, saturated solutions of ISN and ISN/HP-β-CD were prepared, and their water solubility was analyzed. ISN, known for its poor water solubility, was found to have a solubility of approximately 0.5088 mg/mL. However, upon forming the inclusion complex with HP-β-CD, the solubility increased significantly to 3.6550 mg/mL, resulting in a clear solution. This demonstrates the effectiveness of the HP-β-CD complexation approach in improving the solubility of ISN and achieving a more soluble formulation.

#### 3.2.3. Morphological Studies of ISN/HP-β-CD Inclusion Complex

**Figure 4:**
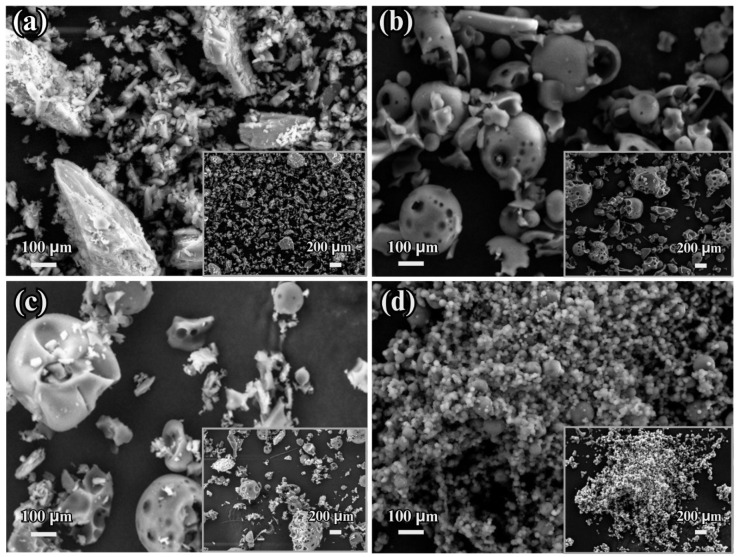
SEM Images of (**a**) pure ISN, (**b**) pure HP-β-CD, (**c**) physical mixture of ISN and HP-β-CD, and (**d**) ISN/HP-β-CD inclusion complex at 100 µm magnification. Insets show 200 µm magnification.

The surface morphology of ISN, HP-β-CD, their physical mixture (PM), and the ISN/HP-β-CD inclusion complex was analyzed using scanning electron microscopy. Figure 4 presents the SEM images of (a) pure ISN, (b) pure HP-β-CD, (c) the physical mixture of ISN and HP-β-CD, and (d) the ISN/HP-β-CD inclusion complex.

Pure ISN (Figure 4a) exhibited irregularly shaped particles with a semi-crystalline structure, characterized by a rough surface and uneven particle sizes. HP-β-CD (Figure 4b) displayed a spherical morphology with characteristic cavities, indicative of its porous molecular structure. The physical mixture (PM) of ISN and HP-β-CD (Figure 4c) displayed a combination of the morphologies of the individual components, indicating no significant interaction between the drug and cyclodextrin molecules in this state.

In contrast, the ISN/HP-β-CD inclusion complex (Figure 4d) exhibited a distinct transformation compared to its individual components. The particles appeared chaotic and somewhat spherical, with the disappearance of the crystalline structure of ISN and the porous cavities of HP-β-CD. These pronounced morphological changes can be attributed to the successful formation of the inclusion complex, where ISN is encapsulated within the hydrophobic cavity of HP-β-CD. This encapsulation results in molecular rearrangement and an amorphous appearance, supporting the enhanced solubility and stability of ISN within the HP-β-CD matrix.

#### 3.2.4. Thermal Analysis of ISN/HP-β-CD Inclusion Complex

**Figure 5:**
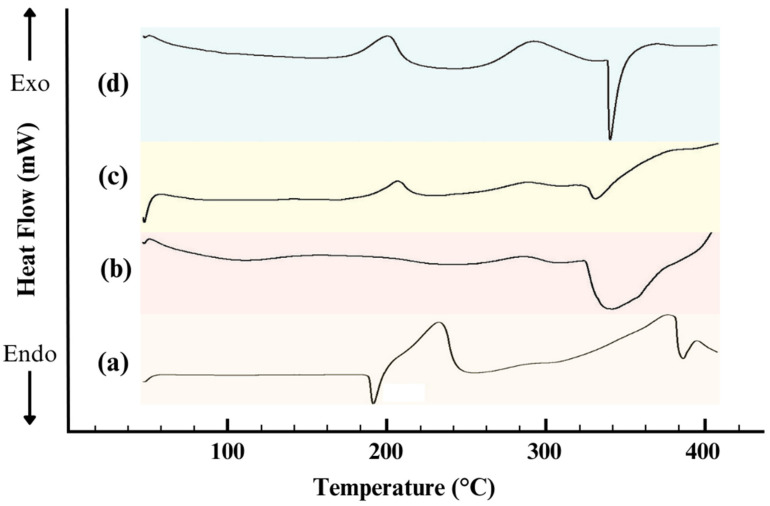
DSC thermograms of (**a**) pure ISN, (**b**) pure HP-β-CD, (**c**) ISN/HP-β-CD physical mixture (PM), and (**d**) ISN/HP-β-CD inclusion complex.

Differential Scanning Calorimetry (DSC) is an essential technique for investigating the thermal properties and phase behavior of drug-polymer systems [24,25]. The DSC thermograms of pure ISN, HP-β-CD, ISN/HP-β-CD physical mixture (PM), and ISN/HP-β-CD inclusion complex are presented in Figure 5. The thermogram of pure ISN exhibited a sharp endothermic peak at 187.5 °C, indicative of its crystalline nature. In contrast, HP-β-CD displayed two broad endothermic peaks: one at ~80 °C, corresponding to the loss of bound water molecules, and another at ~270 °C, associated with its thermal degradation, confirming its amorphous nature.

The physical mixture retained both the ISN melting peak and the dehydration peak of HP-β-CD, though the ISN peak was slightly broader and shifted. This suggests minor interactions but no significant complex formation, indicating that simple mixing does not induce strong molecular interactions. However, the DSC thermogram of the ISN/HP-β-CD inclusion complex showed a complete disappearance of the ISN melting peak, suggesting successful encapsulation of ISN within the HP-β-CD cavity and a transition from a crystalline to an amorphous state [26,27]. This structural transformation is advantageous for improving solubility and bioavailability, as amorphous forms typically exhibit enhanced dissolution properties.

The DSC results confirm that the ISN/HP-β-CD inclusion complex has a distinct thermal profile, characterized by the absence of the ISN melting peak and the presence of broad thermal events associated with HP-β-CD. These findings indicate strong molecular interactions between ISN and HP-β-CD, supporting the successful formation of the inclusion complex. This transformation enhances the thermal stability and pharmaceutical performance of ISN, making the complex a promising formulation for improved drug solubility and bioavailability.

#### 3.2.5. FT-IR Analysis of ISN/HP-β-CD Inclusion Complex

**Figure 6:**
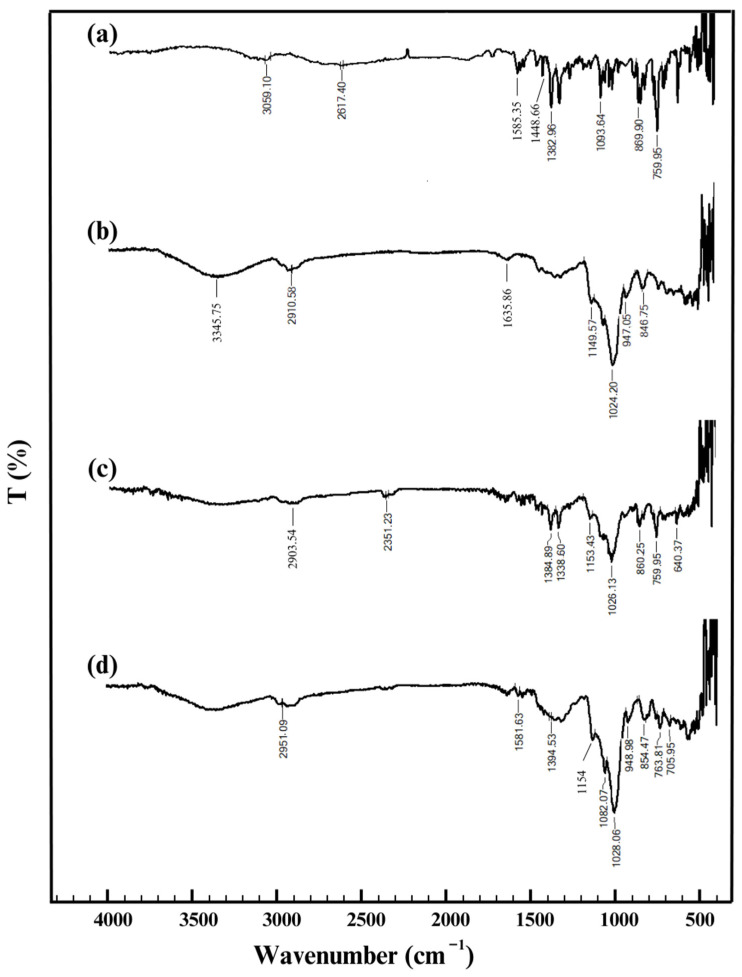
FT-IR spectra of (**a**) pure ISN, (**b**) pure HP-β-CD, (**c**) physical mixture of ISN and HP-β-CD, and (**d**) ISN/HP-β-CD inclusion complex.

FT-IR spectroscopy was conducted to investigate the molecular interactions between Isoconazole Nitrate (ISN) and Hydroxypropyl-β-Cyclodextrin (HP-β-CD) by comparing the spectra of pure ISN, HP-β-CD, their physical mixture (PM), and the inclusion complex (Figure 6). Key functional groups were identified based on characteristic absorption bands.

The FT-IR spectrum of ISN displayed a broad peak at 3059 cm^−1^, corresponding to aromatic C-H stretching, along with a peak at 2617 cm^−1^, which may be an overtone or combination band. The absorption at 1585 cm^−1^ was attributed to C=C stretching in the aromatic rings, while the bands at 1449 cm^−1^ and 1382 cm^−1^ were associated with NO_3_^−^ asymmetric and symmetric stretching, confirming the presence of the nitrate functionality [28,29]. Additional peaks at 1094 cm^−1^ corresponded to C-O-C stretching, while those at 870 cm^−1^ and 760 cm^−1^ were attributed to C-Cl stretching, indicating the presence of ether and chlorinated aromatic groups. These findings align with previous reports on Isoconazole, as obtained by Drabińska et al. [30].

The FT-IR spectrum of HP-β-CD exhibited a characteristic broad band at 3345 cm^−1^, corresponding to O-H stretching, indicative of an extensive hydrogen bonding network. The peaks at 2911 cm^−1^ represented C-H stretching, while the absorption at 1636 cm^−1^ was attributed to H-O-H bending, signifying the presence of water molecules. The absorption bands at 1150 cm^−1^ and 1024 cm^−1^ corresponded to C-O stretching and C-O-C stretching, respectively, characteristic of the ether bonds in the glycosidic structure. Peaks at 947 cm^−1^ and 847 cm^−1^ were associated with ring vibrations and glycosidic linkage vibrations, confirming the presence of glucopyranose units. These results are consistent with those reported in the literature [31,32,33].

The FT-IR spectrum of the physical mixture (PM) revealed an overlay of peaks from both ISN and HP-β-CD, indicating their coexistence without significant molecular interactions (Figure 6c). The peak at 2904 cm^−1^ was assigned to C-H stretching, while the absorption at 2351 cm^−1^ likely corresponded to atmospheric CO_2_ absorption or overtone vibrations. The NO_3_^−^ symmetric and asymmetric stretching bands appeared at 1385 cm^−1^ and 1339 cm^−1^, confirming the presence of ISN. The C-O-C stretching vibrations of HP-β-CD were observed at 1153 cm^−1^ and 1026 cm^−1^, while the C-Cl stretching vibrations from ISN appeared at 880.25 cm^−1^ and 760 cm^−1^. Additionally, the peak at 640 cm^−1^ was associated with out-of-plane bending vibrations. The preservation of these distinct peaks without significant shifts or intensity changes suggests that only physical mixing occurred, with ISN retaining its molecular integrity and HP-β-CD remaining structurally unmodified.

The FT-IR spectrum of the inclusion complex (Figure 6d) demonstrated key changes indicative of successful complexation between ISN and HP-β-CD. The NO_3_^−^ symmetric stretching band at 1394 cm^−1^ was weakened, suggesting an interaction between the nitrate group of ISN and the cyclodextrin cavity. Additionally, the C-Cl stretching peaks from ISN (949 cm^−1^, 863 cm^−1^, and 768 cm^−1^) showed reduced intensity, further supporting ISN’s encapsulation within HP-β-CD. The C-O-C stretching bands of HP-β-CD at 1154 cm^−1^ and 1028 cm^−1^ remained intact, confirming the structural preservation of the cyclodextrin. These spectral shifts, along with the disappearance of ISN-specific peaks, confirm the formation of the inclusion complex, which enhances ISN’s solubility and stability, making it a promising formulation for improving its pharmaceutical properties. The observed changes are consistent with established literature on host-guest interactions in cyclodextrin complexes [34].

#### 3.2.6. ^1^H-NMR Analysis of ISN/HP-β-CD Inclusion Complex

**Figure 7:**
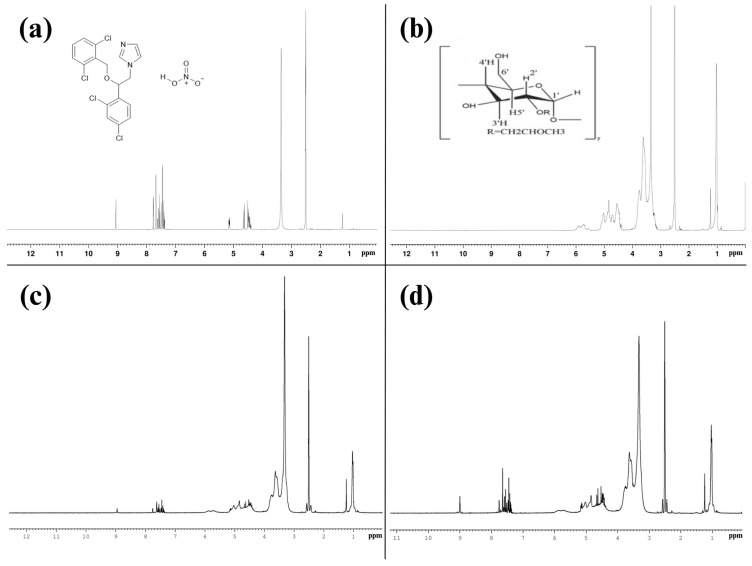
^1^H-NMR spectra of (**a**) pure ISN, (**b**) pure HP-β-CD, (**c**) physical mixture of ISN and HP-β-CD, and (**d**) ISN/HP-β-CD inclusion complex.

The ^1^H NMR spectra of ISN, HP-β-CD, their physical mixture (PM), and the inclusion complex (ISN/HP-β-CD) provide critical insights into the molecular interactions between ISN and HP-β-CD. A comparative analysis of chemical shifts (δ), peak intensities, and the disappearance or broadening of specific signals confirms the formation of an inclusion complex (Figure 7).

The ^1^H NMR spectrum of ISN presents well-defined peaks, with aromatic protons (Ar-H) appearing in the 7.3–9.0 ppm range, corresponding to the benzene and imidazole rings. The benzylic methylene (-CH_2_-) protons adjacent to the oxygen atom appear between 5.10 and 5.70 ppm, while ether (-OCH_2_-) protons resonate in the 3.1–4.5 ppm region. The solvent signal from DMSO-d_6_ is visible at 2.50 ppm, with a residual water peak around 3.3 ppm. These signals confirm the structural integrity of ISN (Figure 7a).

HP-β-CD exhibits characteristic glucose ring proton signals, with chemical shifts for H-1, H-2, H-3, H-4, H-5, and H-6 at 4.985, 3.520, 3.969, 3.418, 3.762, and 3.867 ppm, respectively. These values match previously reported data for HP-β-CD and indicate a well-preserved cyclodextrin structure (Figure 7b) [35,36]. The importance of H-3 and H-5 lies in their involvement in host-guest interactions, making them key binding sites in inclusion complexes [37,38]. The absence of additional peaks confirms the purity and stability of HP-β-CD in DMSO-d_6_.

The ^1^H NMR spectrum of the physical mixture of ISN and HP-β-CD contains peaks from both individual components, confirming the presence of unbound ISN and HP-β-CD in the mixture (Figure 7c). The ISN peaks remain prominent, and no significant chemical shift changes are observed in HP-β-CD protons. This indicates that there is no strong interaction or inclusion occurring between ISN and HP-β-CD in the physical mixture. Only minor intensity variations are noticed, which are likely due to dilution effects and intermolecular interactions rather than true complexation.

In contrast to the PM, the ^1^H NMR spectrum of the inclusion complex reveals significant spectral changes (Figure 7d). The ISN peaks appear broadened or reduced in intensity, indicating encapsulation inside the HP-β-CD cavity. This is attributed to molecular shielding effects caused by host-guest interactions. The most notable chemical shift changes occur at H-3 and H-5, shifting from 3.969 ppm to 3.9655 ppm and 3.762 ppm to 3.7595 ppm, respectively. These changes, reflected in Δδ_1_ values, confirm that the interaction is localized at these specific binding sites.

Table 3 presents the chemical shift differences (Δδ_1_ and Δδ_2_) for HP-β-CD in the PM and complex. The largest shifts at H-3 (−0.0035 ppm) and H-5 (−0.0025 ppm) confirm that these protons participate in the binding interaction with ISN. Conversely, H-1, H-2, H-4, and H-6 exhibit negligible changes, reinforcing that the inclusion occurs selectively at H-3 and H-5. The PM spectrum lacks these shifts, further proving that true complexation only occurs in the inclusion system.

The ^1^H NMR analysis suggests the formation of the ISN/HP-β-CD inclusion complex. The selective chemical shift changes at H-3 and H-5 and the reduced intensities of ISN peaks indicate molecular encapsulation. Since no significant changes occur in the PM, the findings support that ISN interacts specifically with HP-β-CD through host-guest interactions, aligning with previously reported cyclodextrin inclusion complexes.

### 3.3. In Situ Gel Evaluation: Rheology, pH, and Drug Content

#### 3.3.1. pH Measurement and Gelation Temperature

The pH values and gelation temperatures of the formulated in situ gels were evaluated to determine their suitability for vaginal application (Table 4). The most promising formulations were F12.75H05, which gelled at 33 °C with a pH of 4.04; F14, which gelled at 26 °C with a pH of 4.50; C13.5H05-HPβCD, which gelled at 30 °C with a pH of 4.86; and C14 HPβCD, which gelled at 30 °C with a pH of 4.37. These formulations demonstrated favorable gelation properties, making them strong candidates for vaginal drug delivery.

One of the critical factors affecting the biocompatibility of vaginal formulations is pH, as it plays a significant role in maintaining vaginal homeostasis. The normal vaginal pH typically falls between 3.5 and 4.5, helping to sustain the natural microbial flora and acting as a defense mechanism against infections. Vaginal formulations should align with this pH range to prevent irritation and maintain the physiological balance of the vaginal environment. Additionally, formulations with a compatible pH may help restore vaginal acidity, particularly in individuals experiencing bacterial vaginosis or postmenopausal vaginal atrophy, where pH imbalances can contribute to discomfort and increased susceptibility to infections.

All the tested formulations exhibited pH values within the acceptable vaginal range, indicating their potential suitability for vaginal application. The pH values of these formulations suggest they can be well-tolerated and safe for vaginal administration, ensuring patient comfort and therapeutic effectiveness [39].

#### 3.3.2. Gelation Strength and Retention in Simulated Vaginal Fluid

The in situ gel formulations were designed to undergo gelation at 37 °C, ensuring prolonged retention at the application site and sustained drug release. Maintaining an optimal gel structure is essential, as formulations that dissolve too quickly may lead to rapid drug depletion, while highly viscous gels may hinder effective drug diffusion.

The formulations demonstrated rapid gelation at 37 °C with sufficient strength to sustain controlled drug release. As shown in Table 5, all formulations exhibited a gelling capacity rating of (+++), confirming their stability and suitability for vaginal drug delivery.

These results confirm that the formulated in situ gels have the desired balance between gel strength and viscosity, ensuring effective retention and sustained drug release.

#### 3.3.3. Swelling Kinetics in Simulated Vaginal Conditions

The swelling behavior of the in situ gel formulations was evaluated over a 72 h period using dialysis membranes, which were sealed with a dialysis membrane sealer to maintain controlled conditions. The initial mass of the gel was recorded before the test. The results indicated a gradual increase in swelling over time, showing a proportionate and sustained absorption of fluid.

After 72 h, the swelling percentages were measured as 118.219% for F12.75H05, 184.837% for F14, 195.096% for C13.5H05HPβCD, and 142.156% for C14HPβCD, confirming the gels’ ability to absorb and retain fluid effectively.

#### 3.3.4. Measurement of the Rheological Properties

**Figure 8:**
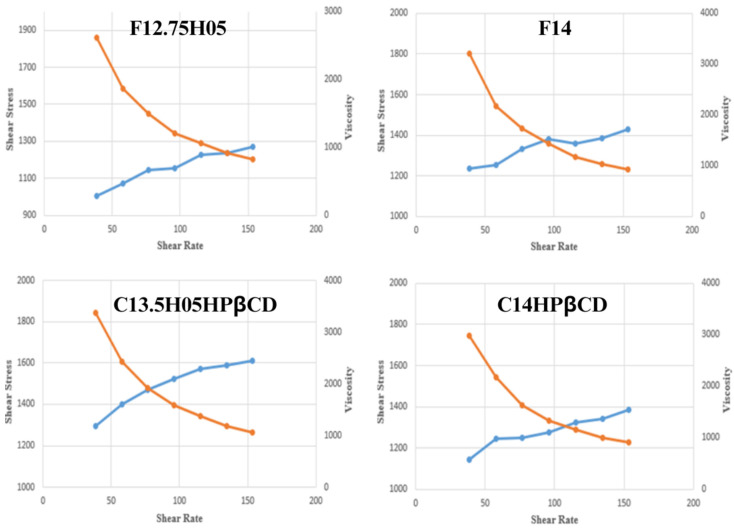
Rheograms of the in situ gel formulations at 25 °C (Day 0) (blue line: shear stress, orange line: viscosity).

**Figure 9:**
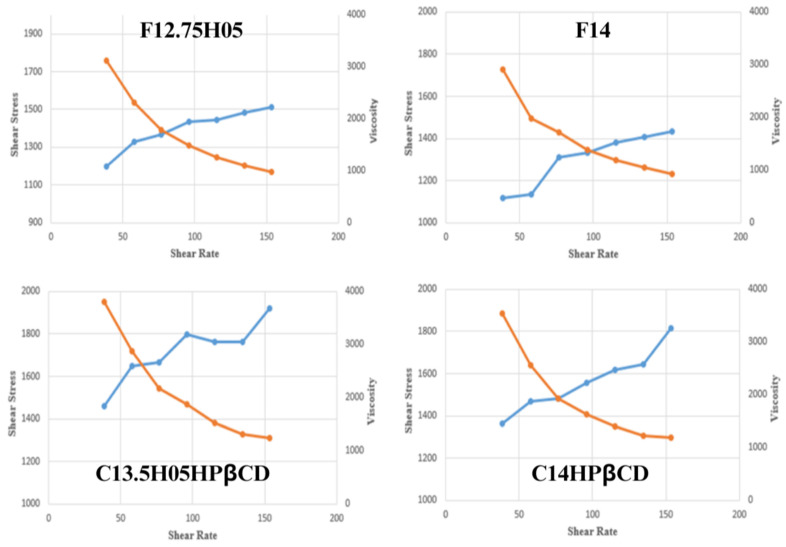
Rheograms of the in situ gel formulations at 37 °C (Day 0) (blue line: shear stress, orange line: viscosity).

The rheological properties of in situ gel formulations play a crucial role in predicting their in vivo performance, particularly for vaginal drug delivery [40]. Figure 8 illustrates the rheograms of the formulations at 25 °C, while Figure 9 shows their behavior at 37 °C, providing insight into their shear stress and viscosity profiles.

At 25 °C, the formulations exhibited a progressive increase in shear stress with increasing shear rate, indicating shear-thinning behavior (Figure 8). This response suggests that the gels remain easily spreadable at room temperature, facilitating their administration and uniform distribution upon application. The viscosity of the formulations decreased as the shear rate increased, which is characteristic of pseudoplastic (non-Newtonian) flow.

At 37 °C, all formulations demonstrated a transition to a gel phase, displaying non-Newtonian pseudoplastic behavior (Figure 9). This transformation is expected for Pluronic^®^-based thermosensitive drug delivery systems, where gelation occurs above the sol–gel transition temperature [41]. The increased viscosity at physiological temperature enhances residence time, ensuring sustained drug release at the application site.

Among the tested formulations, C13.5H05HPβCD exhibited the highest viscosity, followed by C14HPβCD, F12.75H05, and F14. The presence of HPMC contributed to increased mucoadhesion and formulation viscosity, which may improve retention within the vaginal mucosa [39]. Additionally, it has been reported that HPMC-containing formulations exhibit higher elasticity values, further supporting prolonged retention and controlled drug release.

The rheological properties observed in these formulations are crucial for ensuring optimal application, spreadability, and prolonged drug residence time. The higher viscosity and gel strength of the HPβCD-containing formulations suggest their potential for enhanced bioavailability and prolonged therapeutic action.

#### 3.3.5. Determination of Drug Content (DC%)

The drug content of the in situ gel formulations was assessed to ensure uniform distribution and compliance with pharmaceutical standards. ISN and ISN/HP-β-CD inclusion complexes were incorporated into the gels at a 2% ratio, following industry guidelines for vaginal formulations.

The measured drug content values for the formulations are presented in Table 6. The formulations containing pure ISN (F12.75H05 and F14) exhibited drug content values of 0.615% and 0.749%, respectively. Meanwhile, the ISN/HP-β-CD inclusion complex formulations (C13.5H05HPβCD and C14HPβCD) showed drug content values of 0.655% and 0.652%, respectively. Although slightly lower than the theoretical value, these results remain within acceptable pharmaceutical limits for formulation variability. The findings confirm uniform dispersion of the active ingredient within the gel matrices, supporting reproducibility and therapeutic effectiveness [42].

#### 3.3.6. In Vitro Release Studies of In Situ Gel Formulations

**Figure 10:**
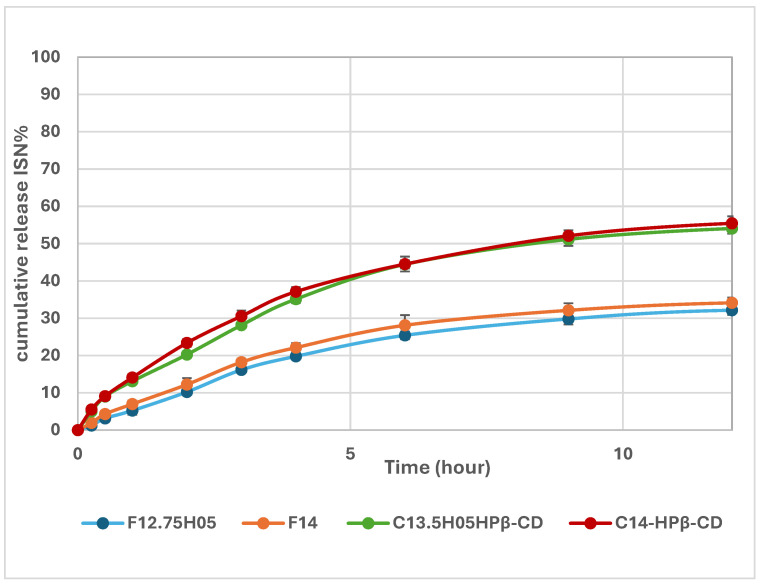
In vitro release profiles of in situ gel formulations containing ISN and ISN/HP-β-CD inclusion complex over 12 h.

The in vitro drug release study plays a critical role in optimizing pharmaceutical formulations. The release profiles of the in situ gel systems are presented in Figure 10. The results indicate that formulations containing ISN/HP-β-CD inclusion complexes (C13.5H05-HPβCD and C14-HPβCD) exhibited a higher drug release rate compared to formulations with pure ISN (F12.75H05 and F14). This enhancement is attributed to the improved water solubility of the inclusion complexes, which facilitates faster drug dissolution and diffusion.

**Figure 11:**
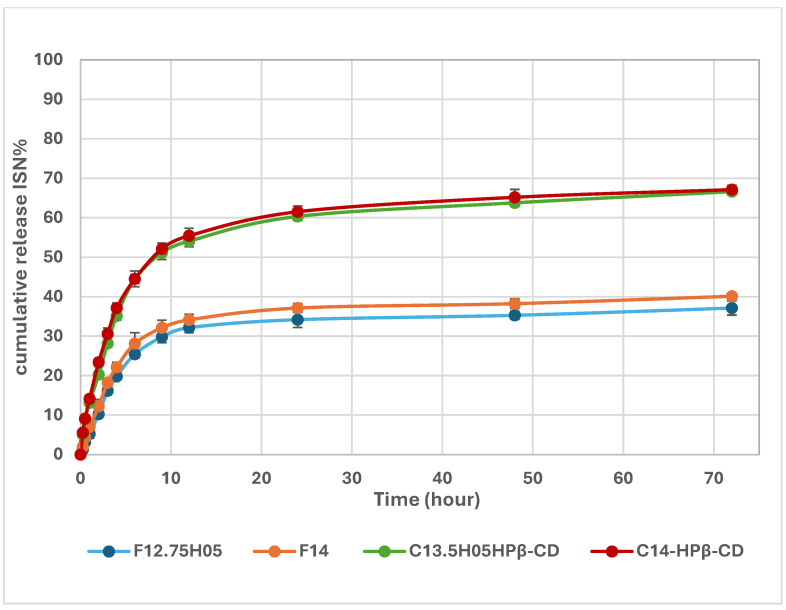
In vitro release profiles of in situ gel formulations containing ISN and ISN/HP-β-CD inclusion complex over 72 h.

In addition to higher solubility, the formulations incorporating inclusion complexes demonstrated a prolonged drug release profile, as illustrated in Figure 11. The ISN/HP-β-CD-loaded gels extended ISN release by approximately 1.5–2 times compared to formulations with pure ISN. This suggests a potential reduction in dosage frequency, which can enhance patient compliance and overall treatment efficacy for antifungal therapy [43].

To further understand the release mechanisms, DD-Solver software (version 1.0) was used to analyze the kinetics of drug release. The release data showed the best fit to the Peppas-Sahlin model, which describes a dual mechanism involving diffusion-controlled (Fickian) release and polymer relaxation-driven (non-Fickian) release. This indicates that the release of ISN from the in situ gel systems is primarily controlled by diffusion, influenced by polymer matrix relaxation [44].

#### 3.3.7. Comparative Antifungal Efficacy Against Candida Strains

The antimicrobial efficacy of the formulated in situ gels was assessed by measuring the inhibition zone diameters after a 24 h incubation period. The study focused on evaluating the antifungal activity of pure ISN-loaded gels and ISN/HP-β-CD inclusion complex-loaded gels against *Candida albicans*, *Candida glabrata*, and *Candida krusei*.

The results, summarized in Table 7, revealed that all tested formulations exhibited antifungal activity, with varying degrees of inhibition depending on the formulation and fungal strain. The highest inhibition zones against *C. glabrata* followed the sequence: C13.5H05HPβCD > F12.75H05 > C14HPβCD > F14. Similarly, the formulations with the most significant inhibition against C. krusei were ranked as C14HPβCD > C13.5H05HPβCD = F12.75H05 > F14. Against C. albicans, the order of effectiveness was observed as C13.5H05HPβCD > F12.75H05 > C14HPβCD > F14.

These results indicate that incorporating HP-β-CD into the formulation did not diminish ISN’s antifungal efficacy; instead, it enhanced its performance against the tested fungal strains. Furthermore, placebo formulations (without ISN) did not exhibit any inhibition zones, confirming that the antifungal activity was solely attributed to ISN and its inclusion complex.

## 4. Conclusions

This study successfully developed in situ gel formulations incorporating isoconazole nitrate (ISN) and its inclusion complex with hydroxypropyl-β-cyclodextrin (HP-β-CD) to enhance solubility, stability, and antifungal efficacy for vaginal drug delivery. The spray-dried inclusion complexes significantly improved the aqueous solubility of ISN, which contributed to its enhanced dissolution and bioavailability. The optimized thermosensitive, mucoadhesive in situ gel formulations, prepared using Pluronic^®^ F127 and hydroxypropyl methylcellulose (HPMC), demonstrated ideal gelation properties, ensuring prolonged retention at the site of action.

The physicochemical characterization confirmed the successful formation of the ISN/HP-β-CD inclusion complex, as evidenced by FT-IR, DSC, SEM, and ^1^H-NMR analyses. The rheological studies further validated the formulations’ pseudoplastic behavior, ensuring ease of application and stability under physiological conditions. Additionally, in vitro release studies indicated a sustained drug release profile, following the Peppas-Sahlin model, suggesting a combined diffusion and relaxation-controlled mechanism.

Most importantly, antimicrobial testing demonstrated superior antifungal efficacy of the ISN:HP-β-CD-loaded gels against *Candida albicans*, *Candida glabrata*, and *Candida krusei*, surpassing the activity of pure ISN formulations. This highlights the potential of cyclodextrin complexation in enhancing drug solubility and therapeutic effectiveness.

Overall, this study provides a promising novel approach for vaginal drug delivery, leveraging cyclodextrin-based inclusion complexation and mucoadhesive in situ gelling systems to optimize ISN’s antifungal activity. These findings pave the way for further clinical evaluation, ensuring a patient-friendly, effective, and long-acting treatment for vaginal infections.

## Figures and Tables

**Table 1:** Components of optimal formulations (*w*/*w* %).

Formulation Code	Active Substance (%)	Pluronic^®^ F127 (%)	HPMC(%)	Benzalkonium Chloride (%)	Distilled Water (%)
F12.75H05	1	12.75	0.5	0.01	To 100 mL
F14	1	14	-	0.01	To 100 mL
C13.H05HPβCD	1	13.5	0.5	0.01	To 100 mL
C14HPβCD	1	14	-	0.01	To 100 mL

**Table 2:** Gelling capacity classification.

Observation	Coding
No gelation	-
Gelation occurred in a few minutes and remained for few hours	+
Gelation was immediate and remained for a few hours	++
Gelation was immediate and for an extended period	+++
Very stiff gel	++++

**Table 3:** Chemical shift perturbations of HP-β-CD in different formulations.

Proton	Complex (δ)	PM (δ)	HP-β-CD (δ)	Δδ_1_ (Complex − (HP-β-CD))	Δδ_2_ (PM − (HP-β-CD))
H-1	4.9870	4.9865	4.985	0.0020	0.0015
H-2	3.5218	3.5215	3.520	0.0018	0.0015
H-3	3.9655	3.9680	3.969	−0.0035	−0.0010
H-4	3.4200	3.4195	3.418	0.0020	0.0015
H-5	3.7595	3.7610	3.762	−0.0025	−0.0010
H-6	3.8658	3.8662	3.867	−0.0012	−0.0008

**Table 4:** pH measurements of in situ gel formulations containing pure ISN and ISN/HP-β-CD inclusion complex.

Content	Code	pH
Pure ISN	F12.75H05	4.04
Pure ISN	F14	4.50
ISN/HP-β-CD Complex	C13.5H05HPβCD	3.86
ISN/HP-β-CD Complex	C14HPβCD	4.37

**Table 5:** Gelling capacity of in situ gel formulations.

Formulation Code	Gelling Capacity
F12.75H05	+++
F14	+++
C13.H05HPβCD	+++
C14HPβCD	+++

**Table 6:** Drug content of in situ gel formulations.

Formulation Code	Drug Content (mg) ± SE * (*n* = 3)
F12.75H05	0.615 ± 0.044
F14	0.749 ± 0.096
C13.H05HPβCD	0.655 ± 0.142
C14HPβCD	0.652 ± 0.253

* SE: Standard error.

**Table 7:** Inhibition zone diameters for antimicrobial tests of in situ gel formulations.

Microorganism			
	*C. albicans*	*C. glabrata*	*C. krusei*
Formulation Code	Zone Diameter mm		
F12.75H05	39.66	39.00	37.66
C13.5H05HPβCD	40.00	42.33	38.33
F14	33.66	33.66	35.66
C14HPβCD	37.33	38.33	40.66
Standard deviation	2.42	2.61	1.94

## Data Availability

The data presented in this study are available in this article.

## Abbreviations

The abbreviations used in this manuscript are as follows:

ISN	Isoconazole Nitrate
HP-β-CD	Hydroxypropyl-β-cyclodextrin
SEM	Scanning Electron Microscopy
FT-IR	Fourier Transform Infrared Spectroscopy
^1^H-NMR	Proton Nuclear Magnetic Resonance
DSC	Differential Scanning Calorimetry
HPMC	Hydroxypropyl Methylcellulose
CDs	Cyclodextrins
SVF	Simulated Vaginal Fluid
HPLC	High-Performance Liquid Chromatography
PM	Physical Mixture
DMSO-d_6_	Deuterated Dimethyl Sulfoxide
rpm	Revolutions Per Minute
DC%	Drug Content Percentage
DD Solver	Drug Dissolution Solver Program
K1:1	Stability Constant for 1:1 Drug–CD Complex
Ks	Stability Constant
R^2^	Correlation Coefficient
δ	Chemical Shift (for NMR spectroscopy)
ppm	Parts per Million
Ar-H	Aromatic Hydrogen
